# EasyDock 1.3: An Automated Pipeline for Molecular Docking

**DOI:** 10.1021/acs.jcim.6c01221

**Published:** 2026-06-11

**Authors:** Guzel Minibaeva, Veincent Yap, Pavel Polishchuk

**Affiliations:** 1 Institute of Molecular and Translational Medicine, Faculty of Medicine and Dentistry, Palacky University, Hněvotínská 1333/5, Olomouc 779 00, Czech Republic; 2 54761 Nanyang Technological University, 50 Nanyang Ave, Singapore 639798, Singapore

## Abstract

Molecular docking is widely used in drug design, particularly for large compound libraries. We previously developed EasyDock, an automated docking pipeline with multiserver task distribution to support such large-scale campaigns, and present here its extended version. Supported docking engines now include Vina-family CPU- and GPU-based variants (QVina2, Vina-GPU, etc.) and deep-learning-based engines (CarsiDock, SurfDock) via a built-in client–server architecture. Ligand preparation was enriched with salt stripping, stereoisomer enumeration, and conformational sampling of saturated ring systems. Integration of open-source protonation tools (pkasolver, MolGpKa, Uni-p*K*_a_) replaces previously required commercial software, making the pipeline fully open source. Postdocking analysis now includes protein–ligand interaction fingerprint (PLIF) computation and pose quality assessment via PoseBusters. We provide Apptainer/Docker containers for the docking engines and protonation tools, simplifying installation and HPC deployment. The source code is available at https://github.com/ci-lab-cz/easydock.

## Graphical abstract

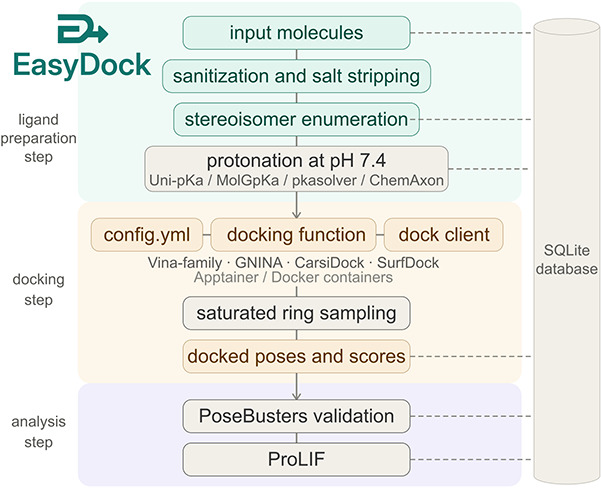

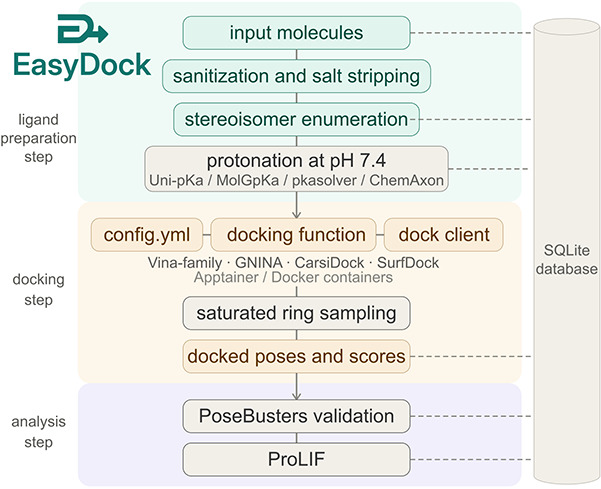

## Introduction

Computational methods have become central to modern drug discovery by streamlining and accelerating the identification of promising molecules.^1–3^ Among them, molecular docking is a key technique for predicting ligand binding poses and affinities, enabling rapid compound prioritization, reducing experimental costs, and supporting virtual screening, lead optimization, and drug repurposing.^3,4^ As computational power, scoring functions, and structural biology continue to advance, its role in modern drug discovery is expected to grow further.

A wide range of open-source docking tools have emerged recently. Empirical-based engines such as AutoDock Vina,^5^ Smina,^6^ QVina2,^7^ and Uni-Dock,^8^ along with CNN-scoring tools like GNINA,^9^ differ primarily in their scoring functions and GPU acceleration. More recently, deep-learning-based approaches including DiffDock,^10^ CarsiDock,^11^ and SurfDock^12^ have introduced, offering alternative paradigms for pose prediction based on generative models and learned representations. Despite this diversity, the practical adoption in large-scale workflows remains hindered by several factors.

First, most tools lack fully automated pipelines covering all preparatory steps: protonation state assignment, stereoisomer enumeration and format conversion. Existing wrappers such as VirtualFlow,^13^ DockStream,^14^ ChemFlow,^15^ and DockString^16^ address some of these steps, but each has some shortcomings. VirtualFlow relies on the commercial ChemAxon cxcalc utility for protonation; DockStream and ChemFlow depend on the Schrödinger’s Epik;^17^ and DockString uses OpenBabel,^18^ whose rule-based protonation is unsuitable for pH-dependent state assignment, especially with multiple or coupled ionizable groups. This dependence on commercial or oversimplified components restricts accessibility and reproducibility, particularly for academic groups without institutional licenses.

Second, deploying modern deep-learning docking tools is challenging. SurfDock, for example, requires specific versions of PyTorch, geometric deep-learning libraries, protein language model embeddings, and surface fingerprint computation, making environment setup error-prone and time-consuming. Many of these tools lack robust containerized distributions, and their integration into automated screening pipelines is often nontrivial. Third, existing pipelines rarely include postdocking analysis, leaving protein–ligand interaction fingerprint (PLIF) computation and pose quality assessment to the user.

We previously developed EasyDock,^19^ a modular open-source pipeline which automates ligand docking except the protein preparation step. Here, we present an extended version. Supported docking engines now include deep-learning-based methods (SurfDock and CarsiDock) and additional Vina-family tools (QVina2, Vina-GPU, etc.) complementing the previous AutoDock Vina support. To streamline such integrations, we developed a client-server architecture in which each docking engine is distributed as an Apptainer/Docker container, simplifying installation, distribution, and HPC deployment; this modular design also allows new engines to be added with relatively little effort. Ligand preparation was extended with open-source protonation via MolGpKa,^20^ pkasolver,^21^ and Uni-p*K*_a_,^22^ removing the need for commercial software. PLIF computation^23^ and PoseBusters-based pose validation^24^ were integrated into the pipeline. EasyDock remains accessible through both a command-line interface and a Python API for straightforward integration into third-party workflows.

## EasyDock Architecture and New Features

EasyDock automates ligand preparation, docking, and postdocking analysis, but does not cover protein preparation. EasyDock is accessible through a command-line interface (*easydock*) and a Python API (*easydock.run_dock.docking*). The screening pipeline is divided into two independent stages: ligand preparation and ligand docking (Figure 1) so that ligands can be prepared once and reused with different docking programs or protein targets without repeating a preparation step.

**Figure 1:**
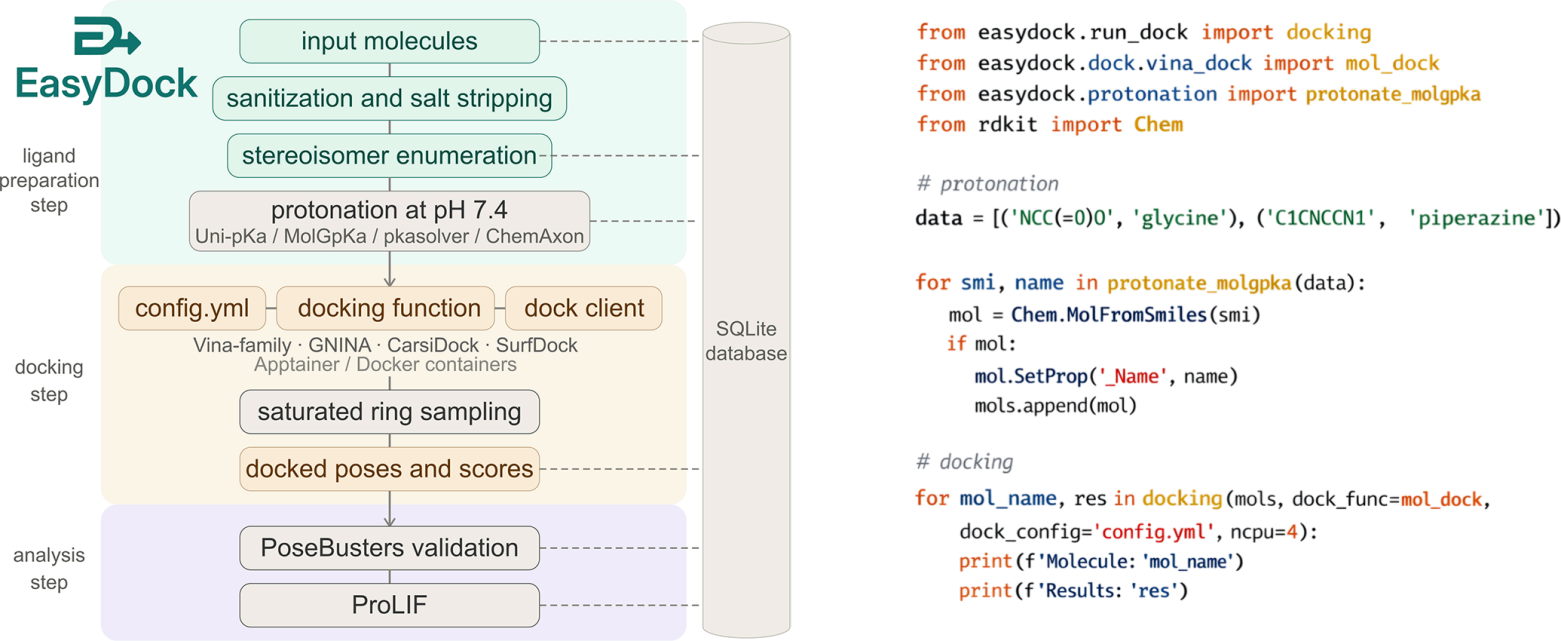
(Left) Overview of the EasyDock pipeline. Gray blocks indicate optional steps. A docking function is imported for each supported program. The config.yml file contains all docking settings. (Right) Example of using the EasyDock Python API for the docking stage.

EasyDock operates through a generator function that takes as input a list of molecules, a docking function wrapping a specific docking program, and a YAML configuration file (config.yml). The configuration file specifies the receptor, docking box coordinates, other program-specific parameters, and therefore varies by docking program. Example configuration files for all supported programs are provided in the repository.

Through its Python API, EasyDock can also be embedded in third-party software. On this basis, we implemented tools for automated *de novo* generation and structure optimization - CReM-dock^25^ (https://github.com/ci-lab-cz/crem-dock) and CReM-opt (https://github.com/ci-lab-cz/crem-opt). EasyDock has also been used in combination with REINVENT^26^ and ChemTS^27^ frameworks for *de novo* molecular generation, demonstrating compatibility with external generative tools.

### Client–Server Architecture for Integration of Docking Engines

EasyDock implements a lightweight client-server architecture based on interprocess communication to support docking programs with complex runtime dependencies, including deep-learning engines such as CarsiDock and SurfDock and also GPU-accelerated Vina family tools. This design separates the EasyDock pipeline from each program runtime environment, allowing engines to run in self-contained Apptainer/Docker containers without exposing dependencies to the host. It also separates one-time initialization from the repeatedly executed docking step.

The protocol uses JSON strings (one JSON object per line) sent through a subprocess’s standard input and output. The EasyDock client starts the docking server as a long-lived child process and communicates with it using three commands: *info*, *init*, and *dock*. *info* retrieves server defaults, such as ligand and output pose formats (PDBQT, MOL, SDF, etc.) and preferred batch size. *init* sends docking settings, including receptor and auxiliary file paths, to initialize the program once at startup and avoid repeated setup. *dock* sends a batch of ligands and returns docking results with predicted binding scores and poses.

To minimize overhead, a single server process is kept alive within a worker thread for the duration of a screening campaign; the connection is automatically re-established if the server process terminates unexpectedly. On the server side, each supported engine implements the same three-command interface, allowing new docking programs to be integrated via a minimal server script that handles initialization and per-batch docking calls.

Three docking engines were integrated through this protocol, each running as a self-contained server inside its own container. Selection was guided by user requests, with priority given to tools covering different hardware settings (CPU and GPU) so users can match workflows to available resources. Using methods with different scoring functions and modeling strategies also provides more orthogonal predictions, strengthening consensus ranking and confidence in docking results.

CarsiDock^11^ is a deep-learning docking method that predicts protein–ligand distance matrices and converts them into binding poses by geometry optimization. In EasyDock, it takes ligands as SMILES strings. During initialization, the receptor PDB file and a reference ligand defining the binding pocket are provided, and the CarsiDock network and RTMScore model^28^ are loaded into GPU memory once. Ligand conformers are then generated during docking, and the best pose is scored with RTMScore.

SurfDock^12^ is a deep-learning docking method that combines protein sequence, 3D structural graphs, and surface features in an equivariant diffusion model to predict ligand binding poses. In EasyDock, it accepts ligands as MOL blocks. During initialization, the receptor and reference ligand are placed in the required directory structure and preprocessed for surface and pocket features. Ligand batches are then docked by the diffusion model. Returned poses are ranked by pose confidence, and screen confidence is used as the final docking score.

The Vina-family server supports six programs: Vina-GPU, QVina2-GPU, QVinaW-GPU, and their CPU counterparts.^5,7,29,30^ The engine is chosen by a configuration parameter. All use the same scoring function but different search algorithms: QVina2 speeds up Vina with similar accuracy, while QVinaW extends QVina2 with interprocess spatiotemporal integration for blind docking. Ligands are provided in PDBQT format, and each molecule is docked by running the selected binary as a subprocess, with scores and poses extracted from the PDBQT output.

We measured docking runtimes for 819 estrogen receptor 1 ligands from LIT-PCBA^31^ (25 agonists, 794 inactives; Table 1). Although this is a challenging docking benchmark and all programs showed low AUC values, it was suitable for runtime comparison. QVina and Vina were the fastest, but they used 128 CPU cores, whereas the other programs ran on GPU nodes with one A100 GPU and 16 cores. Settings for all programs are provided in the examples directory of the repository. Vina and QVina used 4 cores per molecules, for Gnina the number of cores was set to 1, other programs do not provide control over the number of cores. Vina’s lower CPU utilization (66%) was caused by a few molecules with long docking times (3–5 min) finishing last when most CPUs were idle, which reduced overall statistics. In our experience, screening time scales nearly linearly with the number of compounds, so these timings can be reasonably extrapolated to larger sets, where the effect of a few slow docking molecules is smaller. Final runtimes depend strongly on settings and available hardware and should be optimized individually. In general, the number of molecules docked in parallel (*-c* argument) and the number of cores per molecule (configurable for some programs) should be set so that their product exceeds the available CPU count.

**Table 1:** Screening Run Times for a Set of 819 Ligands of Estrogen Receptor 1 on CPU (128 Cores) and GPU Nodes (16 cores, 1 A100)^a^

Program	Compute node	Docking wall time (h:mm:ss)	CPU utilization	Median docking time of a single ligand	AUC	PoseBusters
Vina	CPU	0:12:30	66%	19.1 s (4 cores)	0.567	99.4%
QVina	CPU	0:05:48	85%	13.9 s (4 cores)	0.565	99.4%
Vina-GPU	GPU	0:31:13	4%	1.7 s	0.558	99.0%
GNINA	GPU	0:41:50	88%	32.9 s	0.587	95.4%
CarsiDock	GPU	0:51:59	65%	3.8 s	0.566	41.1%
SurfDock	GPU	2:15:14	75%	10.1 s	0.559	17.8%

a Vina, QVina and Gnina used exhaustiveness 16.

### Integration of Arbitrary External Docking Programs

To extend EasyDock’s compatibility beyond its natively integrated engines, we implemented an experimental generic interface that allows any external docking program to be incorporated into the pipeline without modifying the core codebase. The interface is configured entirely through a YAML file specifying the executable or Python script, the conda or virtual environment, ligand input/output formats, argument names, and a score extraction pattern. It supports both file- and stream-based I/O, three input representations (PDBQT, SMILES, MOL block), and three output formats (PDBQT, PDB, SDF). Configuration examples and implementation details are provided in Supporting Information.

### Ligand Protonation

The protonation state of ligands affects both the hydrogen bond network formed during docking as well as atom charges, which may be considered by a docking program; these effects collectively influence ligand-protein recognition and the predicted binding affinity.^32,33^ Previously, we incorporated Chemaxon tool, but its accessibility is limited as a commercial product requiring a paid license. To address this, we integrated several open-source tools, including MolGpKa,^20^ pkasolver,^21^ and Uni-p*K*_a_,^22^ demonstrating moderate to high accuracy of p*K*_a_ predictions.^34,35^

pkasolver uses a graph neural network with transfer learning to predict microstate p*K*_a_ values.^21^ It represents the protonated and deprotonated forms of an acid–base pair as molecular graphs, processes them with a GIN-based GNN, and predicts the p*K*_a_ from the paired graph embeddings. The model was pretrained on ChEMBL structures with p*K*_a_ values predicted by Epik, then fine-tuned on experimental p*K*_a_ data. MolGpKa is a graph-convolutional neural network that learns p*K*_a_-relevant chemical patterns automatically from the molecular structure and predicts p*K*_a_ for each ionizable site.^20^ The model was trained on ChEMBL structures with predicted p*K*_a_ by ACD and protonation sites predicted by Epik. Both pkasolver and MolGpKa were integrated into EasyDock through their native Python API.

Uni-p*K*_a_ is a thermodynamics-aware machine-learning framework built on a modified Uni-Mol molecular encoder (a Transformer-based 3D molecular representation model), combined with (i) a microstate enumerator, (ii) a free-energy-based formulation that predicts microstate free energies, and (iii) a module that converts predicted free energies into macro- or micro-p*K*_a_ values while preserving thermodynamic consistency across coupled protonation equilibria.^22^ Uni-p*K*_a_ was trained on ChEMBL structures with p*K*_a_ values predicted by Chemaxon and fine-tuned on experimental data, including DataWarrior pKaInWater^36^ and selected entries from the iBond (https://ibond.las.ac.cn/).

Uni-p*K*_a_ integration required a container to isolate dependencies conflicting with the main EasyDock environment. Several fixes were applied that had not been addressed by the Uni-p*K*_a_ developers in open issues on the GitHub repository. The entire pipeline was substantially refactored to enable parallel execution across multiple CPUs and to support stream-based processing via stdin/stdout pipes. The same containerized approach can be applied to integrate additional protonation tools.

By default, the major microspecies is predicted at pH 7.4. This value can be changed via a command line for all three protonation tools. All tools treat input tautomeric forms explicitly and do not modify them.

Our goal was not a comprehensive comparison of different tools in predicting the major microspecies at pH 7.4, but rather to identify possible discrepancies and notify users about them. As a common denominator, we used predictions from Chemaxon, the only commercial tool accessible to us. For the study, we selected structures from ChEMBL33 with molecular mass of 500 Da or less. Stereochemistry was stripped, as all evaluated tools except Uni-p*K*_a_ operate at 2D level. For Uni-p*K*_a_ a single randomly chosen stereoisomer was used. After duplicate removal 1,748,091 structures remained. Chemaxon (version 24.2.2) was used to obtain reference protonated forms. In total, 807,407 structures were assigned protonation states differed from the initial ones by at least one of the programs (Table 2).

**Table 2:** Comparison of Ligand Protonation State Predictions by Five Tools against Chemaxon (version 24.2.2) as the Reference^a^

Program	Total molecules changed by at least one of the programs	Number of molecules with different protonation states from Chemaxon	Protonation wall time of 10,000 molecules (CPU utilization)
OpenBabel	807,407	278,660 (34.5%)	2 s (1 CPU)
MolGpKa		236,156 (29.2%)	743 s (CPU node) (74%)
			328 s (GPU node) (68%)
MolGpKa fix		209,798 (26%)	782 s (CPU node) (72%)
			306 s (GPU node) (75%)
pkasolver fix		378,257 (46.8%)	171 s (CPU node) (48%)
Uni-p*K*_a_		172,323 (21.3%)	1781 s (CPU node) (93%)
			834 s (GPU node) (77%)

a Dataset: ChEMBL structures with molecular weight ≤ 500 Da, restricted to molecules whose protonation state was modified by at least one tool. Runtimes were measured on a random subset of 10,000 molecules. Protonation was run on CPU nodes (128 cores, 256 GB RAM) and GPU nodes (16 CPU cores, A100 GPU, 256 GB RAM).

OpenBabel served as the baseline - a simple, fast, rule-based method. It disregards the mutual influence of ionized groups and therefore tends to overestimate the number of charged states. It produced 278,660 differently protonated molecules relative to Chemaxon.

MolGpKa exhibited a better agreement with Chemaxon (Table 2); nevertheless, 236,156 structures showed protonation states different from the Chemaxon. To address several common discrepancies, we implemented manually crafted rules, which improved consistency. These rules were created specifically to fix protonated centers at pH 7.4 and cannot be applied at other pH values. The full list is provided in Supporting Information. Examples include correcting deprotonation of multiple OH groups within a single ring of polyphenols, overprotonation of closely placed nitrogens, the protonation of amide groups, etc. Since these rules rely on subjective judgment, the original version of MolGpKa is also available via *molgpka* protonation argument, while the fixed version is invoked via *molgpka_fix.*

pkasolver showed substantially more discrepancies relative to Chemaxon. For this reason, we incorporated only one obvious fix – reverting protonated nitrogens in amide/sulfonamide groups to their neutral states, enabled by default for all pkasolver predictions.

Among the evaluated methods, Uni-p*K*_a_ demonstrated the best agreement with Chemaxon, with 172,323 structures showing differing protonation states. To characterize these discrepancies, we performed a functional group-level analysis (see Supporting Information). We identified several groups that Chemaxon systematically deprotonated while Uni-p*K*_a_ did not, including N-acetyl-N-arylamine (cNC­(C)=O), N′-acetyl aroylhydrazide (cC­(=O)­NNC­(C)=O), diaroylhydrazine (cC­(=O)­NNC­(c)=O), etc. A number of discrepancies also arose from competing protonation sites with close p*K*_a_ values. Uni-p*K*_a_ results were reproducible for most molecules because a fixed seed is used for conformer embedding. However, the predicted protonation states for 13,376 (1.7%) out of 793,431 compounds having multiple stereoisomers were different for different stereoisomers indicating sensitivity of Uni-p*K*_a_ to 3D structures. Multiple runs also revealed that 108 compounds yielded different protonation states for some stereoisomers across runs. The list of these stereoisomers is given in Supporting Information.

### Protein–Ligand Interaction Fingerprints

The binding mode of a docked ligand can provide valuable information for compound prioritization, particularly in lead optimization and when selecting compounds expected to recapitulate interactions observed in a reference cocrystal structure.^37–39^ To support this, EasyDock integrates protein–ligand interaction fingerprint (PLIF) computation based on the ProLIF library.^23^

Ten interaction types are detected for each docked pose: hydrophobic contacts, hydrogen bond donation and acceptance, cationic and anionic interactions, cation−π and π–cation contacts, face-to-face and edge-to-face π–π stacking, and metal coordination. The resulting binary fingerprint encodes the presence or absence of each interaction with each protein residue are stored in the EasyDock SQLite database, supporting incremental calculation and later retrieval without reprocessing.

A key practical feature is similarity scoring against a user-defined reference fingerprint, typically derived from a known active compound or a cocrystallized ligand. Similarity is computed as the fraction of reference interactions reproduced by the docked pose, ignoring additional contacts not present in the reference. This allows compounds to be filtered or ranked by their ability to reproduce the pharmacophoric interactions of interest, complementing the docking score as an orthogonal selection criterion.

### Pose Validation with PoseBusters

A known limitation of deep learning-based molecular docking approaches, is that high-ranking poses may contain physically implausible features - steric clashes with the protein, distorted bond geometries, or violations of fundamental chemical rules - that are invisible to the scoring function but would be rejected on physical grounds upon manual inspection. These artifacts arise from the simplifications inherent in docking search algorithms and scoring functions. Filtering such poses before downstream analysis reduces the risk of pursuing false positives.

EasyDock integrates PoseBusters,^24^ a rule-based pose validation tool that applies geometry, chemistry, and protein–ligand compatibility checks to docked structures. Validation is performed in the *dock_fast* mode, which evaluates ligand geometry and clashes with the receptor without a cocrystal reference structure. Results – a pass/fail flag per molecule and pose – are stored in the EasyDock database, enabling their use as filters at the retrieval stage. Molecules can be extracted from the database with an option to retain only those that passed all PoseBusters checks, enabling one-step filtering. As with PLIF computation, PoseBusters validation is performed incrementally: previously processed molecules are skipped on subsequent runs, making the check practical for large databases in stages.

### Other Features

Ligand preparation was extended with salt stripping and stereoisomer enumeration. Salt stripping is performed with the RDKit SaltRemover, which removes counterions and small fragments commonly present in compound library records. Molecules remaining multicomponent after salt removal are flagged and excluded from docking. The original unmodified SMILES is retained in the database alongside the processed structure to ensure traceability.

For molecules supplied as 2D structures or SMILES having undefined chiral centers or double bonds, stereoisomers are enumerated using RDKit EnumerateStereoisomers, with the number of generated isomers controlled by a user-defined parameter. Each stereoisomer receives a unique stereo ID and is treated as an independent entity: stored separately and docked independently. Molecules provided as 3D SDF structures bypass stereoisomer enumeration, and their input geometry is used directly for docking. Some complex molecules require considerable time to generate physically plausible 3D structures; if plausible 3D coordinates cannot be generated within 300 s, enumeration is interrupted and the molecule is skipped.

For docking programs requiring 3D coordinates for input ligands, starting conformers are generated with RDKit’s ETKDGv3 algorithm followed by MMFF94 force field minimization. To improve docking of compounds with saturated rings systems in programs treating rings as rigid bodies, multiple input conformers can be generated optionally. Up to 50 initial conformers are generated, clustered by RMSD of ring atoms and their directly bonded neighbors using agglomerative clustering. A representative subset of structurally diverse conformers is retained. Each conformer is submitted to docking independently, and all poses from the best-scoring across all input conformers are reported.

We evaluated the performance of ring sampling in redocking on a set of 1410 protein–ligand complexes from the refined subset of PDBbind (version 2020)^40^ containing at least one saturated ring system of up to 8 atoms. Redocking was performed using Vina and GNINA. In both cases, ring sampling increased the number of successfully redocked structures, defined as poses within 2Å RMSD of the X-ray reference. While the absolute differences were small (Figure S1), the paired *t*-test showed the statistical significance of the improvements (Table S2). The computational runtime increased approximately 3-fold due to the docking of additional starting conformers, that should be considered when applying ring sampling to large-scale virtual screening.

Other enhancements include more detailed logging and documentation. The logging level is controlled via a command line argument; setting it to DEBUG mode produces additional messages that help identify the source of potential issues. The documentation covers not only installation and usage with examples but also includes a developer section describing internal conventions and interfaces (https://easydock.readthedocs.io/en/latest/).

To extract docked poses and scores, a command line utility *get_sdf_from_easydock* retrieves selected poses as an SDF file or returns structures as SMILES together with docking scores.

Interrupted screenings (due to system failures, timeouts, or user intervention) can be resumed by restarting the same command; results are committed incrementally to the SQLite database, and completed molecules are automatically skipped.

## Limitations

Protein structure preparation and docking grid box definition are not integrated in EasyDock, as curation of protein structures may be nontrivial and often requires manual intervention. We recommend preparing the protein manually and carefully inspecting the structure, in particular for missing side chains or residues and misplaced hydrogen atoms (especially on histidines), since such issues affect the entire docking campaign.

All EasyDock features are available on Linux and macOS systems. On Windows, only docking programs with native Windows binaries (for example, a standalone Vina binary) can be run by substituting *script_file* with a Windows binary in the configuration. Containerized features can be run via Docker, although EasyDock on Windows has not been thoroughly tested.

The client-server docking protocol does not support distributed virtual screening, which was implemented in the previous EasyDock version via the Dask library.^41^ Dask setup was found to be relatively complex, therefore we are looking for a more convenient alternative rather than reimplementing Dask support. The previously integrated docking engines, however, can still use it.

## Future Enhancements

Currently, docking results for the same ligand set are stored in separate databases for different proteins or docking programs. In future versions, we plan to combine all results for a given molecule set into a single database to facilitate consensus predictions and better assessment of ligand selectivity. We also aim to add pose rescoring with alternative docking programs as a preliminary step before more computationally intensive methods such as MM-GBSA/PBSA^42–44^ or free energy perturbation.^45^ We are also considering the incorporation of more advanced protonation prediction tools that may improve accuracy at the cost of longer computational times. This would be particularly beneficial for smaller compound libraries, where extended protonation runtimes remain manageable.

## Conclusions

The current version of EasyDock provides an automated, open-source pipeline covering all stages of ligand-protein docking except protein preparation. The pipeline is accessible via both the command line and a Python API. Its client-server architecture enables seamless integration with multiple docking engines, substantially broadening the range of supported tools compared to the previous version. Newly added engines include deep-learning-based methods (CarsiDock and SurfDock) as well as additional Vina-family tools (QVina2, Vina-GPU, QVina2-GPU), extending the GNINA and AutoDock Vina support already present in the earlier release. Several open-source protonation tools were integrated and evaluated; among them, Uni-p*K*_a_ demonstrated the highest concordance with the commercial ChemAxon software. Other key additions include protein–ligand interaction fingerprint (PLIF) computation for downstream pose analysis, PoseBusters-based pose validation, and sampling of saturated ring conformations to improve the accuracy of docking poses. EasyDock is accompanied by comprehensive documentation covering installation, usage, advanced workflows, analysis, and customization.

## Supplementary Material





## Data Availability

The source code of EasyDock is available under the BSD-3 license at https://github.com/ci-lab-cz/easydock. The third-party integrated protonation and docking tools are distributed under Apache 2.0 or MIT licenses, with the exception of Meeko, which is licensed under the LGPL-2.1 license. The validation data for protonation tools are available in the Zenodo repository at 10.5281/zenodo.17179256. Prebuilt docking and protonation containers are available at 10.5281/zenodo.19652220 and 10.5281/zenodo.17506577.

## Notes

Additional details of integration of arbitrary external docking programs with examples; the list of SMARTS patterns used to fix MolGpKa outputs; detailed statistics on computational performance of protonation tools and discrepancies in ligand protonation patterns between Uni-p*K*_a_ and Chemaxon; performance and statistics of docking using ring sampling (PDF)
Performance and statistics of docking using ring sampling (ZIP)
