# Supplementary material for: EasyDock 1.3: An Automated Pipeline for Molecular Docking
Source: J Chem Inf Model. 2026 Jun 11;66(15):8691–7. doi: 10.1021/acs.jcim.6c01221 (PMC13471725; doi:10.1021/acs.jcim.6c01221)
Supplement: Supplementary file 1 [file ci6c01221_si_001.pdf]

EasyDock 1.3: an automated pipeline for molecular docking

Guzel Minibaeva[1], Veincent Yap[2], Pavel Polishchuk[1]

[1] Institute of Molecular and Translational Medicine, Faculty of Medicine and Dentistry, Palacky University, Hněvotínská 1333/5, 779 00 Olomouc, Czech Republic
[2] Nanyang Technological University, 50 Nanyang Ave, 639798 Singapore, Singapore

Supplementary materials

Integration of arbitrary external docking programs

The generic interface enables arbitrary external docking programs to be incorporated into EasyDock without modifying its core codebase. Configuration is provided entirely through a YAML file that specifies the path to the executable or Python script, the conda or virtual environment in which it should be invoked, the ligand input and output formats, the argument names, and a score extraction pattern.

Input/output handling. The interface supports both file-based and stream-based I/O. When explicit input and output argument names are provided, ligand structures are written to temporary files and passed to the binary/script via command-line arguments; otherwise, the ligand is piped to the program via standard input, and the docking pose is read from standard output, avoiding disk I/O overhead. Three input representations (PDBQT, SMILES, and MOL block) and three output formats (PDBQT, PDB, and SDF) are supported, covering the conventions of most available docking programs. Scores are extracted from the output using either a named SDF property field or a user-defined regular expression applied to the raw output block, providing flexibility for programs with non-standard output formats. An example configuration file is given below; further examples are available in the online documentation.

Environment resolution. For Python-based programs requiring isolated dependencies, the appropriate interpreter is resolved automatically: if the env configuration parameter points to a directory, the Python binary within that environment is used directly; if it specifies a named environment, the program is launched via conda run, with automatic detection of conda, mamba, or micromamba. This mechanism enables programs with complex or conflicting dependency stacks, such as deep-learning models, to be run from dedicated environments without manual path management.

Limitations. The generic interface does not separate initialization and docking phases like in the case of client-server docking integration, making it inefficient for programs with time-consuming initialization (model loading, etc.), as this overhead is incurred for each batch. The current implementation also assumes that the program processes one structure at a time, which is not the case for engines capable of handling multiple structures in parallel. We therefore expect this interface to evolve in future releases.

An example of a yaml-configuration file to run QVina2 as a separate executable using generic interface

```
script_file: ~/qvina/bin/qvina2.1
ligand_in_format: pdbqt
ligand_out_format: pdbqt
input_arg_name: --ligand
output_arg_name: --out
score_mode: min

program_args:
  receptor: ~/2btr/A/2btr_A.pdbqt
  config: ~/2btr/grid.txt
  exhaustiveness: 8
  num_modes: 5
  cpu: 2
  seed: 0

parse_score:
  score_regex: REMARK VINA RESULT:\s+(-?[\d.]+)
```

MolGpKa patterns which revert protonated sites back to neutral state

Two-point centers: among two-point centers one which had less favorable pK_a predicted value was reverted to a neutral state

```
[#7+;!$([#7+][O-]);!$([#7+H0v4])]~*~[#7+;!$([#7+][O-]);!$([#7+H0v4])]
[#7+;!$([#7+][O-]);!$([#7+H0v4])]**[#7+;!$([#7+][O-]);!$([#7+H0v4])]
[#7+;!$([#7+][O-]);!$([#7+H0v4])]***[#7+;!$([#7+][O-]);!$([#7+H0v4])]
c[n-][n-]c
[O-]cc[O-]
[O-]cac[O-]
[O-]caac[O-]
```

One-point centers; the charged center was reverted to a neutral state

```
[$([NH+,NH2+,NH3+]-[*]=[O,S])]
[$([N-](C(=O))[N-]S(=O)=O)]
[$([N-]1C(=O)N(C)C(=O)C1)]
[$([NH-](C(=O))C1=NC=CS1)]
[$([NH2+]C(=[NH+])[NH2])]
[$([N-]C(=[NH+])[NH2])]
[$([nH+]1aaaa1)]
[$([n-]1c(=O)[n-]c(=O)cn1)]
[$([n-]1ncnc1[N-]S(=O)=O)]
[$([O-]aaC(=O)[O-])]
[$([#8-][#6][#7-])]
[$([O-][CX4][CX3][O-])]
[$([N+]=C[N-]S(=O)=O)]
[$([n-]1cnc(=O)c2cccn12)]
[$([N-](a)C([NH2])=[NH2+])]
[$([NX4+;H1,H2,H3]c)]
[$([O-][CX4])]
```

Table S1. Comparison of protonation state predictions by five tools against Chemaxon (version 24.2.2) as the reference. Dataset: ChEMBL structures with molecular weight ≤ 500 Da, restricted to molecules whose protonation state was modified by at least one tool. Runtimes were measured on a randomly chosen subset of 10,000 molecules. Protonation was run on CPU nodes (128-CPU cores, 256 GB RAM) and GPU nodes (16 CPU cores, A100 GPU, 256 GB RAM).

Program	Total molecules changed by at least one of the programs	Number of molecules which were not changed by the program	Number of molecules which were not changed by the program but which were protonated by Chemaxon	Number of molecules which were changed by the program	Number of molecules which were changed by the program but which protonation states were differed from Chemaxon	Number of molecules which had different protonation states for the program and Chemaxon	protonation time of 10000 molecules (CPU utilization)
OpenBabel	807,407	241,741	96,702	565,666	181,958	278,660	2 s (1 CPU)
MolGpKa	807,407	286,202	90,903	521,205	145,253	236,156	743 s (CPU node) (74%)<br>328 s (GPU) (68%)
MolGpKa fix	807,407	291,262	92,880	516,145	116,918	209,798	782 s (CPU node) (72%)<br>306 s (GPU) (75%)
pkasolver fix	807,407	215,641	104,387	591,766	273,870	378,257	171 s (CPU node) (48%)
Uni-pKa	807,407	314,689	90,709	492,718	81,614	172,323	1781 s (CPU node) (93%)<br>834 s (GPU node) (77%)
Chemaxon	807,407	257,682	0	549,725	0	0	-

Table S2. The number of docked ligands and reproduced poses in re-docking depending on the initial set of conformers.

Initial conformer(s) used for docking	Program	The number of ligands with redocking poses having RMSD ≤ 2 to the reference pose	The number of successfully docked ligands	p-value (Student test)	Cumulative run time (hours)
The lowest conformer	Vina 1.2.7	655 (47.1%)	1,391	0.037	15.67
The lowest conformer + ring sampling		714 (51.3%)	1,391		46.22
The lowest conformer	Gnina 1.3	918 (65.1%)	1,410	**$1.3*10^{-4}$**	41.67
The lowest conformer + ring sampling		978 (69.4%)	1,410		132.18

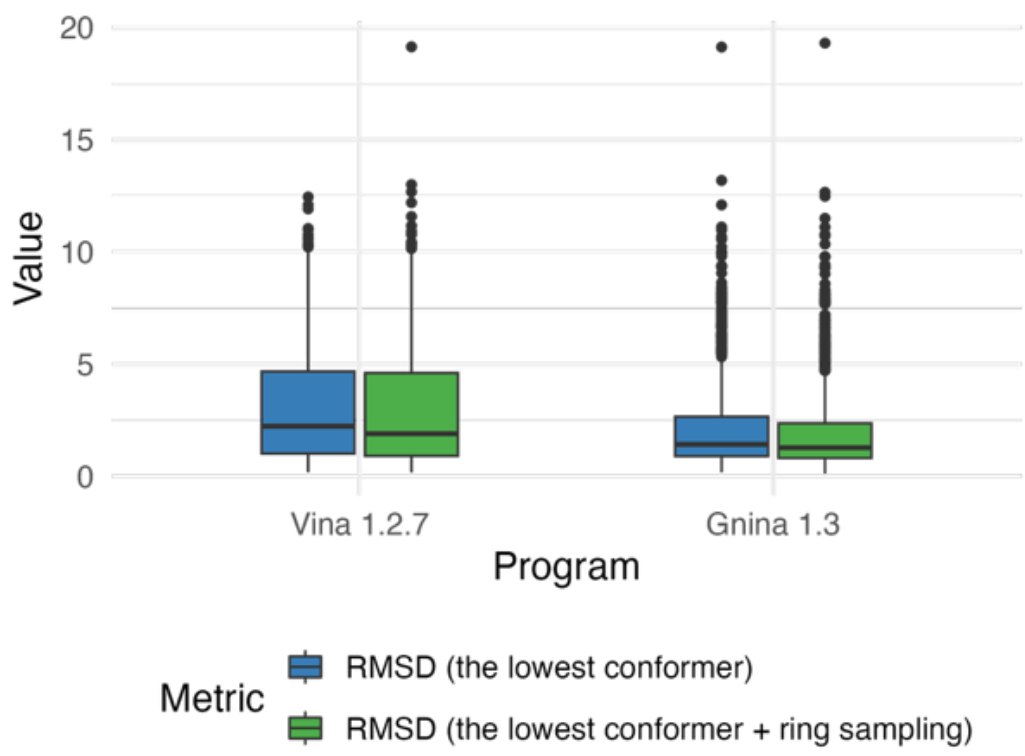

Figure S1. RMSD between X-ray reference structures and docked poses obtained for cases using the lowest energy conformer as a starting one and a set of representative conformers defined based on saturated ring conformations.

Analysis of discrepancies of protonated functional groups

To identify systematic differences in protonation state predictions between ChemAxon cxcalc and Uni-pKa at the functional group level, the following analysis was performed. For each molecule, ionizable atoms were identified by matching Dimorphite-DL SMARTS patterns [3], and functional groups were extracted using the IFG algorithm [1, 2]. Charge states assigned by both tools were then compared for each ionizable atom and aggregated by functional group type. A disagreement ratio was computed for each functional group class to reveal which chemotypes are most susceptible to inconsistent protonation predictions between the two methods.

Table S2 presents a comprehensive overview of protonation state disagreements between ChemAxon and Uni-pKa across all ionizable functional groups identified in ChEMBL compounds. The disagreement rate varies substantially by functional class, ranging from near-complete concordance to 100% discordance, indicating that the two algorithms diverge not uniformly but in a structurally specific manner.

Tertiary amines (CN(C)C) represent the largest single population, with 204,902 individual protonation predictions and a disagreement rate of 24.74% (50,699 mismatches across 48,325 unique molecules), making this the most impactful source of inter-tool inconsistency. Among the 36,203 cases where ChemAxon protonated but Uni-pKa did not, Uni-pKa left the molecule unchanged in 71.7%, produced a different modification in 24.8%, and returned no prediction in 3.5%. In the 15,776 reverse cases, ChemAxon left the molecule unchanged in 65.8% and produced a different modification in 34.2%. Overall, 53.4% of unique molecules were exclusively protonated by ChemAxon, 21.4% exclusively by Uni-pKa, and 6.7% were modified by both tools but in non-identical ways. These results indicate that the majority of disagreements arise from one tool protonating a nitrogen that the other leaves entirely neutral, rather than from both tools selecting competing protonation sites within the same molecule.

Phenols (cO) showed 19,029 individual predictions with disagreements across 17,627 unique molecules. Among the 11,555 cases where ChemAxon deprotonated but Uni-pKa did not, Uni-pKa left the molecule unchanged in 60.7%, produced a different modification in 39.2. In the 7,474 reverse cases where Uni-pKa deprotonated but ChemAxon did not, ChemAxon left the molecule unchanged in 68.9% and produced a different modification in 31.1%. Overall, 38.5% of unique molecules were exclusively deprotonated by ChemAxon, 26.9% exclusively by Uni-pKa, and 2.7% were modified by both tools in non-identical ways.

Table S3. The most frequently occurrent patterns which have different protonation states in Chemaxon and Uni-pKa protonated structures. Patterns are encoded by SMILES where the first atoms in SMILES strings is a charged center.

pattern			chemaxon / uni-pka charge							total occurrence	number of patterns having different charges in Uni-pKa and Chemaxon	fraction of patterns having different charges in Uni-pKa and Chemaxon
type	atoms	element	-1/-1	-1/0	1/-1	1/1	1/0	0/-1	0/1			
CN(C)C	N	N				154203	34923		15776	204902	50699	24.74
cnc	n	N	102	137		6644	17704	54	2629	27270	20524	75.26
cO	O	O	15454	11545				7474		34473	19019	55.17
CNC	N	N				73441	7268		2884	83593	10152	12.14
c[nH]c	[nH]	N	2295	3914		47	108	2751	51	9166	6824	74.45
cNS(c)(=O)=O	NS(=O)=O	N	8818	5239		N		1265		15322	6504	42.45
CN	N	N				40415	5329		453	46197	5782	12.52
cNC(C)=O	NC=O	N		4218						4218	4218	100
cC(=O)NNC(C)=O	O=CNNC=O	N		1682						1682	1682	100
cC(=O)NNC(c)=O	O=CNNC=O	N		1652						1652	1652	100
cn[nH]c	n[nH]	N	1129	890			1	559		2579	1450	56.22
cNC(=O)Nc	NC(N)=O	N		1219						1219	1219	100
cNC	N	N		18		4	18		1017	1057	1053	99.62
cNS(C)(=O)=O	NS(=O)=O	N	892	857				179		1928	1036	53.73
cNc	N	N		285			12		614	911	911	100
cC=C1SC(=O)NC1=O	C=C1SC(=O)NC1=O	N		885						885	885	100
cN(C)C	N	N				7	154		701	862	855	99.19
c[nH][nH]c	[nH][nH]	N	73	682				164		919	846	92.06
c=N	N				15	715	420	61	286	1497	782	52.24
cNC(c)=O	NC=O	N		644				3		647	647	100
CN=C(N)OC	N=C(N)O	N				4	571			575	571	99.3
cC(=N)N	N=CN	N				4361	545		14	4920	559	11.36
c-n(c)[nH]c	[nH]n	N	245	538				19		802	557	69.45
cC(C)=NO	C=NO		6	541						547	541	98.9
cC(=O)NNS(c)(=O)=O	O=CNNS(=O)=O	N		514						514	514	100

cC=C1SC(=S)NC1=O	C=C1SC(=S)NC1=O	N		510						510	510	100
cC=NO	C=NO		108	416				54		578	470	81.31
NC1=NCCO1	N=C(N)O	N				340	409			749	409	54.61

References:

1. Ertl, P. Cheminformatics Analysis of Organic Substituents. *J. Chem. Inf. Comput. Sci.* **2003**, 43(2), 374–380. https://doi.org/10.1021/ci0255782
2. RDKit: Open-source cheminformatics. https://www.rdkit.org;
IFG: https://github.com/rdkit/rdkit/tree/master/Contrib/IFG
3. Ropp, P.J. et al. Dimorphite-DL: an open-source program for enumerating the ionization states of drug-like small molecules. *J. Cheminform.* **2019**, 11, 14. https://doi.org/10.1186/s13321-019-0336-9
